# Empirical Antibiotic Prescribing in Adult COVID-19 Inpatients over Two Years in Mexico

**DOI:** 10.3390/antibiotics11060764

**Published:** 2022-06-02

**Authors:** Efrén Murillo-Zamora, Xóchitl Trujillo, Miguel Huerta, Oliver Mendoza-Cano, José Guzmán-Esquivel, José Alejandro Guzmán-Solórzano, María Regina Ochoa-Castro, Alan Gabriel Ortega-Macías, Andrea Lizeth Zepeda-Anaya, Valeria Ruiz-Montes de Oca, Mónica Ríos-Silva, Agustin Lugo-Radillo

**Affiliations:** 1 Departamento de Epidemiología, Unidad de Medicina Familiar No. 19, Instituto Mexicano del Seguro Social, Av. Javier Mina 301, Col. Centro, Colima 28000, Mexico; efren.murilloza@imss.gob.mx; 2 Facultad de Medicina, Universidad de Colima, Av. Universidad 333, Col. Las Víboras, Colima 28040, Mexico; jose.esquivel@imss.gob.mx (J.G.-E.); jguzman6@ucol.mx (J.A.G.-S.); mochoa24@ucol.mx (M.R.O.-C.); a00344177@tec.mx (A.G.O.-M.); azepeda4@ucol.mx (A.L.Z.-A.); 3 Centro Universitario de Investigaciones Biomédicas, Universidad de Colima, Av. 25 de julio 965, Col. Villas San Sebastián 28045, Mexico; rosio@ucol.mx (X.T.); huertam@ucol.mx (M.H.); 4 Facultad de Ingeniería Civil, Universidad de Colima, km. 9 carretera Colima-Coquimatlán, Coquimatlán 28400, Mexico; 5 Unidad de Investigación en Epidemiología Clínica, Hospital General de Zona No. 1, Instituto Mexicano del Seguro Social, Av. Lapislázuli No. 250, Col. El Haya, Villa de Álvarez 28984, Mexico; 6 Escuela de Medicina, Plantel Guadalajara, Universidad Cuauhtémoc, Av. del Bajío No. 5901, Col. Del Bajío, Zapopan 45019, Mexico; vruiz@ucgdl.edu.mx; 7 Centro Universitario de Investigaciones Biomédicas, CONACyT-Universidad de Colima, Av. 25 de julio 965, Col. Villas San Sebastián, Colima 28045, Mexico; 8 CONACYT—Faculty of Medicine and Surgery, Universidad Autónoma Benito Juárez de Oaxaca, Col. Ex Hacienda de Aguilera S/N, San Felipe del Agua, Oaxaca 68020, Mexico

**Keywords:** COVID-19, SARS-CoV-2, inpatients, anti-bacterial agents, drug prescriptions

## Abstract

*Background and Objectives:* Empirical antibiotic prescribing in patients with coronavirus disease 2019 (COVID-19) has been common even though bacterial coinfections are infrequent. The overuse of antibacterial agents may accelerate the antibiotic resistance crisis. We aimed to evaluate factors predicting empirical antibiotic prescribing to adult COVID-19 inpatients over 2 years (March 2020–February 2021) in Mexico. *Materials and Methods*: A cross-sectional analysis of a nationwide cohort study was conducted. Hospitalized adults due to laboratory-confirmed COVID-19 were included (*n* = 214,171). Odds ratios (OR) and 95% confidence intervals (CI), computed by using logistic regression models, were used to evaluate factors predicting empirical antibiotic prescribing. *Results:* The overall frequency of antibiotic usage was 25.3%. In multiple analysis, the highest risk of antibiotic prescription was documented among patients with pneumonia at hospital admission (OR = 2.20, 95% CI 2.16–2.25). Male patients, those with chronic comorbidities (namely obesity and chronic kidney disease) and longer interval days from symptoms onset to healthcare seeking, were also more likely to receive these drugs. We also documented that, per each elapsed week during the study period, the odds of receiving antibiotic therapy decreased by about 2% (OR = 0.98, 95% CI 0.97–0.99). *Conclusion:* Our study identified COVID-19 populations at increased risk of receiving empirical antibiotic therapy during the first two years of the pandemic.

## 1. Introduction

Empirical prescription of antibiotics in hospitalized patients with coronavirus disease 2019 (COVID-19) has been a frequent event since the pandemic started in late 2019. The medical reasoning supporting this decision in COVID-19 patients is to treat for possible community-acquired bacterial pneumonia and the difficulty in distinguishing between bacterial and virus-related symptoms, given that both of them cause unspecific symptoms such as coughing and fever [1].

The burden of the COVID-19 pandemic in Mexico has been high. By the end of March 2022, more than 5.6 million cases had been confirmed together with 320,000 related deaths [2]. Currently, there is no scientific evidence to support the widespread empirical prescription of antibiotics in COVID-19 inpatients [3]. This results from the low frequency of laboratory-proved bacterial infections in these patients, which ranges from 6–15% [4,5].

The use of antibiotics changes the intestinal microbiota and compromises the immunity of patients with COVID-19 [6]. In addition, long-term use of antibiotics causes resistance of existing bacteria and old strains that are resistant to most antibiotics [7].

Despite this, published data suggest that during the first pandemic wave antibiotics were prescribed to about 7 out of 10 adults hospitalized due to COVID-19 [8]. To the best of our knowledge, there are no published studies assessing the frequency of the prescription of antibiotics to COVID-19 inpatients in Mexico, where high levels of antimicrobial resistance were documented before the recent pandemic [9]. This study aimed to identify, over a time framework of two years in Mexico, factors determining the risk of empirical antibiotic prescribing in hospitalized adults with laboratory-confirmed COVID-19.

## 2. Materials and Methods

A cross-sectional analysis of a retrospective cohort study was conducted in Mexico. Hospitalized adults (aged 20 years or older) with laboratory-confirmed (reverse transcription-polymerase chain reaction [RT-PCR] in nasopharyngeal swab) COVID-19, and illness onset from March 2020 to February 2022, were eligible. The RT-PCR testing (SuperScript™ III Platinum™ One-Step RT-PCR Kits) was performed according to normative standards [10]. The molecular diagnosis took place in one of the four laboratories specialized in epidemiological surveillance that the Mexican Institute of Social Security (IMSS, the Spanish acronym) has throughout the country. A broader description of the methods of the cohort study from where the participants were extracted was published elsewhere [11].

Eligible subjects were patients that were hospitalized in secondary or tertiary healthcare settings and were identified from the nominal records of a normative online system for the epidemiology surveillance of respiratory viral pathogens which belongs to the Mexican Institute of Social Security (IMSS, the Spanish acronym). Patients with incomplete data, as well as those that were only confirmed by rapid antigenic testing, were excluded.

The main binary (no/yes) outcome was the empirical prescription of any antibiotic and it was defined by the ordering of any antibacterial agent at hospital admission. These data, and the specific antibiotic that was ordered, were retrieved from the audited surveillance system. Primary sources of this system are the medical files and death certificates, when applicable.

Other clinical and epidemiological data of interest were also retrieved from the audited surveillance system. Collected data included: demographic characteristics; date of symptoms onset; date of healthcare-seeking; date of hospital admission; clinical and radiographic findings at entry. Pneumonia patients (no/yes) were those with clinical (fever, cough, and dyspnea) and radiographic findings (ground-glass opacities in CT scanning or X-ray) suggestive of this abnormality [12].

Summary statistics were computed. We used unconditional logistic regression models to estimate odds ratios (OR) and 95% confidence intervals (CI) to evaluate factors predicting antibiotic prescription. All analyses were conducted using Stata version 16.0 (StataCorp; College Station, TX, USA).

## 3. Results

**Figure 1:**
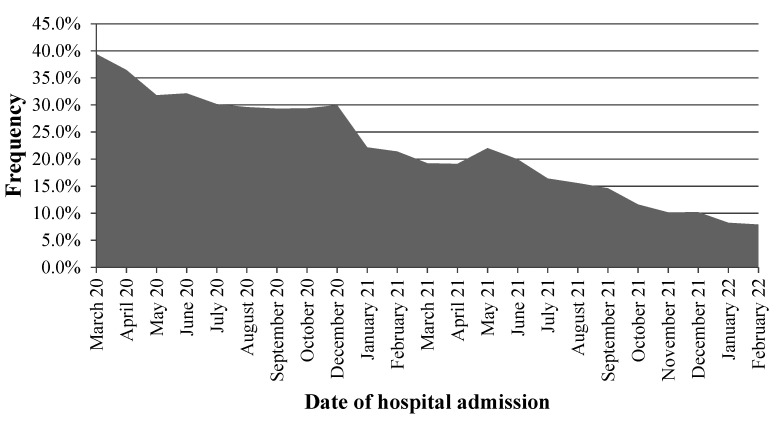
Frequency of empirical antibiotic prescription in inpatients with laboratory-confirmed COVID-19, Mexico 2020–2022.

Data from 214,171 inpatients were analyzed. The overall prevalence of empirical antibiotic prescription was 25.3% (n = 54,208) and the monthly frequency ranged from 39.4% to 7.9% (Figure 1). Cephalosporins were the most ordered drugs (75.7%), followed by macrolides (13.8%), and penicillins (5.5%). The mean age (±standard deviation) of participants was 59.0 ± 15.5 years old and most of them (58.7%) were males.

As presented in Table 1, hospitalized patients for whom empirical antibiotics were prescribed were more likely to be male (59.9% vs. 40.1%, p < 0.001), to be diagnosed with pneumonia at hospital entry (55.4% vs. 44.6%, p < 0.001), and to have had a longer interval from symptoms onset to healthcare seeking (5.5 ± 3.7 vs. 4.9 ± 3.8 days, p < 0.001). The prevalence of obesity (body mass index of 30 or higher) and chronic kidney disease (any stage) was also higher in patients with antibiotics prescription.

In the multiple analysis (Table 2), pneumonia diagnosis at hospital entry was associated with a two-fold increase in the odds of antibiotic prescribing (OR = 2.20, 95% CI 2.16–2.25). Male patients (OR = 1.03, 95% CI 1.01–1.05), those with a longer interval from symptoms onset to healthcare seeking (vs. 3 days or under: 4–7 days, 1.54, 95% CI 1.51–1.58; 8 or above, OR = 1.55, 95% CI 1.51–1.59), and those presenting chronic comorbidities (namely obesity [OR = 1.04, 95% CI 1.02–1.07)] and chronic kidney disease [OR = 1.16, 95% CI 1.12–1.21]), were at increased risk of receiving antibiotics. We also observed a protective effect of the pandemic development on antibiotics usage, and each additional week (counted from the start of the pandemic in Mexico) decreased the odds of antibiotics prescription by about 2% (OR = 0.98, 95% CI 0.97–0.99).

## 4. Discussion

Our study identified several factors predicting the empirical prescription of antibiotics to adult inpatients with laboratory-confirmed COVID-19 during the first two years of the pandemic in Mexico. The presented results suggest that the frequency of prescribing antibacterial drugs has progressively decreased during the analyzed period. The observed scenario may be determined, at least partially, by the accumulation of scientific data regarding the management of COVID-19 inpatients and the availability of rapid antigenic testing.

In general, the observed frequency of empirical antibiotic prescribing in hospitalized patients in our analysis was lower than in others previously published in economically developed populations (which ranged from 57%–60%) [13,14]. This was documented even during the first six months of the COVID-19 pandemic in Mexico when antibacterial drugs were prescribed to about one third of hospitalized patients (32.9% total range: 27.5% to 48.5%).

The hospitals where the patients from our study received medical attention were all public settings (350 and 36 secondary and tertiary healthcare settings, respectively) belonging to the IMSS. The IMSS is an employer-based health scheme that provides health and social services to more than 83 million people in Mexico, which represents about 64% of the total population of the country [15]. We hypothesize that this may be determining, at least partially, the observed difference, since a higher risk of receiving antibiotics has been documented in for-profit hospitals [13].

Antibiotic resistance is a public health threat that was accelerated by the excessive use of antibiotics in both clinical and community settings in the effort to treat COVID-19. The negative implications are immediate. A recently published metagenomics analysis of fecal samples of COVID-19 patients who received empirical antibiotics, which were compared with healthy controls and with patients who did not receive them, evidenced that antibacterial agents increased the abundance of antibiotic-resistant genes (ARGs) in the intestinal flora and altered the composition of ARG profiles [16].

In general, male patients, those with comorbid conditions, and those with more severe symptoms, were at increased odds of receiving any antibacterial drug. We hypothesize, given the predictors of severe illness that were documented early during the pandemic, that patients with any of these characteristics were more likely to present more severe symptoms and therefore to receive empirical antibiotic therapy [17,18].

Published studies evaluating the effectiveness of antibiotics in COVID-19 patients have mainly focused on the use of azithromycin [19,20]. The results are inconsistent and even higher mortality, due to arrhythmia, has been documented among patients in whom this macrolide was administered [21]. Macrolides were prescribed to about 14% of participants in our study, however, most of these drugs (>90%) corresponded to clarithromycin.

We analyzed a large set of individuals and we proceeded to evaluate the effect size in the estimates of interest obtained through the regression model. We computed omega-squared (ω^2^) and most of the estimators were ≤0.01, therefore the effect size might be considered small. A more significant effect was observed for “pneumonia” (ω^2^ = 0.027, 95% CI 0.026–0.029) but it is still small [22]. Therefore, the presented estimates are significant in terms of effect size.

The potential limitations of our study must be discussed. First, we lacked follow-up data that would allow us to determine which of the analyzed patients had a laboratory-proved bacterial coinfection. Second, a small number of comorbidities were analyzed and we lacked data regarding other common chronic conditions (i.e., type 2 diabetes mellitus) that may be also determining the analyzed event. And third, the exposition to antibiotics was analyzed as monotherapy and we were unable to identify patients with two or more antibacterial agents.

## 5. Conclusions

The empirical prescription of antibiotics in adult inpatients with COVID-19 was a common event in the analyzed settings and during the first two years of the pandemic. We identified populations at increased risk of receiving antibacterial agents before confirmation of bacterial coinfection.

## Figures and Tables

**Table 1:** Characteristics of the study sample for selected variables, Mexico 2020–2022.

Characteristic	Overall		Antibiotic Was Prescribed				*p*
	(*n* = 214,171)		No (*n* = 159,963)		Yes (*n* = 54,208)		
**Sex**							
Female	88,376	(41.3)	66,636	(41.7)	21,740	(40.1)	<0.001
Male	125,795	(58.7)	93,327	(58.3)	32,468	(59.9)	
**Age (years)**							
**Age group (years)**							
20 to 39	27,695	(12.9)	21,173	(13.2)	6522	(12.0)	<0.001
40 to 59	80,243	(37.5)	59,251	(37.0)	20,992	(38.7)	
60 or above	106,233	(49.6)	79,539	(49.8)	26,694	(49.2)	
**Dominant variant at illness onset**							
Ancestral	170,414	(79.6)	122,555	(76.6)	47,859	(88.3)	<0.001
Delta (B.1.617.2)	35,581	(16.6)	29,963	(18.7)	5618	(10.4)	
Omicron (B.1.1.529)	8176	(3.8)	7445	(4.7)	731	(1.4)	
**Days from symptoms onset to healthcare seeking**							
3 or less	84,933	(39.7)	67,208	(42.0)	17,725	(32.7	<0.001
4 to 7	76,262	(35.6)	54,303	(34.0)	21,959	(40.5	
8 or above	52,976	(24.7)	38,452	(24.0)	14,524	(26.8)	
**Pneumonia at hospital admission**							
No	126,877	(59.2)	102,713	(64.2)	24,164	(44.6)	<0.001
Yes	87,294	(40.8)	57,250	(35.8)	30,044	(55.4)	
**In-hospital outcome**							
Recovery	109,437	(51.1)	83,165	(52.0)	26,272	(48.5)	<0.001
Death	104,734	(48.9)	76,798	(48.0)	27,936	(51.5)	
*Personal history of:*							
**Obesity (BMI 30 or above)**							
No	172,728	(80.6)	129,853	(81.2)	42,875	(79.1)	<0.001
Yes	41,443	(19.3)	30,110	(18.8)	11,333	(20.9)	
**Chronic kidney disease (any stage)**							
No	199,931	(93.4)	149,552	(93.5)	50,379	(92.9)	<0.001
Yes	14,240	(6.6)	10,411	(6.5)	3829	(7.1)	

Abbreviations: **COVID-19**, Coronavirus disease 2019; **RR**, Risk ratio; **CI**, Confidence interval; **BMI**, Body mass index. Notes: (1) RR and 95% CI were computed by using unconditional logistic regression models; (2) RR and 95% CI from the multiple analysis were adjusted by all the variables listed in the table; (3) Pneumonia was defined by clinical (fever, cough, and dyspnea) and radiographic findings (ground-glass opacities) in CT scanning or X-ray.

**Table 2:** Factors associated with the odds of antibiotic prescription at hospital admission due to laboratory-confirmed COVID-19, Mexico 2020–2022.

Characteristic	OR (95% CI), *p*			
	Bivariate Analysis		Multiple Analysis	
**Sex**				
Female	1.00		1.00	
Male	1.07 (1.05–1.09),	<0.001	1.03 (1.01–1.05),	0.011
**Age group (years)**				
20 to 39	1.00		1.00	
40 to 59	1.15 (1.11–1.19),	<0.001	1.02 (0.99–1.05),	0.259
60 or above	1.09 (1.06–1.12),	<0.001	1.00 (0.97–1.03),	0.962
**Elapsed weeks since the COVID-19 pandemic start in Mexico (per week)**	0.98 (0.97–0.99),	<0.001	0.98 (0.97–0.99),	<0.001
**Days from symptoms onset to healthcare seeking**				
3 or less	1.00		1.00	
4 to 7	1.53 (1.50–1.57),	<0.001	1.54 (1.51–1.58),	<0.001
8 or above	1.43 (1.40–1.47),	<0.001	1.55 (1.51–1.59),	<0.001
**Pneumonia at hospital admission**				
No	1.00		1.00	
Yes	2.23 (2.19–2.28),	<0.001	2.20 (2.16–2.25),	<0.001
*Personal history of:*				
**Obesity (BMI 30 or above)**				
No	1.00		1.00	
Yes	1.14 (1.11–1.17),	<0.001	1.04 (1.02–1.07),	0.001
**Chronic kidney disease (any stage)**				
No	1.00		1.00	
Yes	1.09 (1.05–1.13),	<0.001	1.16 (1.12–1.21),	<0.001

Abbreviations: **COVID-19**, Coronavirus disease 2019; **RR**, Risk ratio; **CI**, Confidence interval; **BMI**, Body mass index. Notes: (1) RR and 95% CI were computed by using unconditional logistic regression models; (2) RR and 95% CI from the multiple analysis were adjusted by all the variables listed in the table; (3) Pneumonia was defined by clinical (fever, cough, and dyspnea) and radiographic findings (ground-glass opacities) in CT scanning or X-ray.

## Data Availability

The data presented in this study are available on request from the corresponding authors.
